# Reduced vessel density in the superficial and deep plexuses in diabetic retinopathy is associated with structural changes in corresponding retinal layers

**DOI:** 10.1371/journal.pone.0219164

**Published:** 2019-07-18

**Authors:** Carlo Lavia, Aude Couturier, Ali Erginay, Bénédicte Dupas, Ramin Tadayoni, Alain Gaudric

**Affiliations:** Ophthalmology Department, Hôpital Lariboisière, AP-HP, Université Paris Diderot - Sorbonne Paris Cité, Paris, France; Massachusetts Eye & Ear Infirmary, Harvard Medical School, UNITED STATES

## Abstract

**Purpose:**

To explore the relationships between vessel density (VD) in the retinal vascular plexuses with the thickness and structural changes of their corresponding retinal layers in patients with diabetic retinopathy (DR).

**Methods:**

Retrospective analysis of 17 eyes of 17 Type 1 diabetes (T1D) patients with severe non-proliferative or proliferative DR and no current or past macular edema. Seventeen age- and sex-matched healthy subjects were included as controls. Using optical coherence tomography (OCT) and OCT-angiography (OCTA), VD was measured in the superficial vascular plexus (SVP) and deep vascular complex (DVC) that includes the intermediate (ICP) and deep capillary plexuses (DCP), and compared to the retinal thickness (RT) of the inner (from the inner limiting membrane to the inner plexiform layer) and intermediate (inner nuclear and outer plexiform layer) retinal layers. The correlation between the inner and intermediate RT and the VD of the corresponding vascular networks (SVP and DVC, respectively) was assessed. All OCT and OCTA examinations were performed using the RTVue XR Avanti (Optovue, Fremont, CA).

**Results:**

The inner RT and VD in all plexuses were significantly reduced in T1D patients compared to healthy subjects. The capillary drop-out patterns were polygonal and well-defined in the SVP while the ICP and DCP showed a more diffuse capillary rarefaction and a VD that varied in the same proportion. The inner RT significantly correlated with VD in the SVP (r = 0.71 in healthy subjects and r = 0.62 in T1D patients, p <0.01). The intermediate RT did not significantly correlate with VD in the DVC.

**Conclusions:**

In T1D subjects, OCTA allowed observing different capillary drop-out patterns in the SVP and in the ICP-DCP, with different structural changes in the corresponding retinal layers, suggesting that they should be considered as distinct anatomical and functional entities.

## Introduction

Macular thickening was the first optical coherence tomography (OCT) parameter found to be correlated with visual acuity (VA) in the context of macular edema (ME) [1,2]. A decrease in macular thickness was then considered to be a good indicator of the efficacy of pharmaceutical treatment of ME [3]. However, on the other hand, an excessive thinning of the macula appears to be also associated with a poorer VA [4,5]. With more detailed retinal structure analysis provided by spectral-domain OCT (SD-OCT), the concept of disorganization of retinal inner layers (“DRIL”) has emerged to characterize cases in which a retinal thinning is associated with the loss of recognizable boundaries between retinal cellular layers in the context of capillary non-perfusion, suggesting some degree of cellular loss [6,7].

Lastly, OCTA implemented with projection artifact removal (PAR) software has shown that, in diabetic retinopathy, capillary drop-out affects the three capillary plexuses of the macula, although VA may remain normal up to a threshold of capillary drop-out which remains to be determined [8,9]. In a previous study, we have confirmed with OCTA the histological finding that the VD of the superficial vascular plexus (SVP) was proportional to the thickness of the corresponding retinal cellular layer in healthy subjects [10]. The aim of this study was to analyze the relationship between the decrease in VD and retinal structural changes in patients with diabetic retinopathy in the absence of documented DME.

## Patients and methods

The research followed the tenets of the Declaration of Helsinki. The study was approved by the Ethics Committee of the French Society of Ophthalmology (IRB 00008855 Société Française d’Ophtalmologie IRB#1). Informed consent was obtained from all patients to allow their records to be reviewed.

### Patients

The records of consecutive type 1 diabetes (T1D) patients diagnosed with severe non-proliferative diabetic retinopathy (NPDR) or proliferative DR (PDR) without DME examined in our tertiary ophthalmological center between April 2015 and April 2017 were retrospectively reviewed.

### Demographics

T1D characteristics (duration and HbA1c level) and comorbidities of patients were recorded. Best-corrected VA (BCVA) was recorded using Snellen charts and then converted into the logarithm of the minimum angle of resolution (logMAR). Age- and sex-matched voluntary healthy subjects were included as a control group. The control group was selected from a larger cohort of healthy subjects matched for age and sex with the patients of the T1D group and did not present any systemic (e.g. hypertension, diabetes, renal failure) or ocular (e.g. glaucoma, macular disease) comorbidities.

### Inclusion and exclusion criteria

General exclusion criteria were: ocular media opacities, clinical evidence of any other retinal disease than DR and prior retinal surgery; a history of pan-retinal photocoagulation (PRP) was not an exclusion criterion.

Inclusion criteria based on OCT and OCTA screening were: no current or past documented DME (central macular thickness [CMT] <275 μm + 2SD and no retinal cysts), preserved retinal structure on structural OCT, no DRIL and good quality OCTA images (signal strength index [SSI] ≥65 and quality index [QI] ≥7). When two eyes were eligible, only the eye showing the higher SSI and QI was included in the analysis.

### Diabetic retinopathy assessment

DR severity was assessed using the Optos scanning laser ophthalmoscope imaging system (Optos PLC, Scotland, UK) according to the simplified American Academy of Ophthalmology DR grading scale [11]. The ETDRS 7-field retinal photo grid was superimposed on Optos images to define DR severity in these fields.

### Optical coherence tomography (OCT) and OCT angiography acquisitions

OCT and OCTA examinations were performed with the RTVue XR Avanti (Optovue, Fremont, CA) spectral-domain OCT device with phase 7 AngioVue software. The OCTA device has a light source at 840 nm, a bandwidth of 45 nm, and an A-scan rate of 70.000 scans per second. A 3-mm x 3-mm macular cube centered on the fovea, composed of 320 horizontal B-scans separated by 9 μm and containing 320 A-scans, was acquired. To correct motion artifacts, OCTA combines orthogonal fast-scan directions (horizontal and vertical) and is equipped with the DualTrac Motion Correction technology [12]. The software includes the 3D PAR algorithm, which removes projection artifacts from the OCTA volume on a per voxel basis using information from the OCT and OCTA volumes to differentiate *in situ* the OCTA signal from projection artifacts [13,14].

### Optical coherence tomography angiography analysis

**Fig 1:**
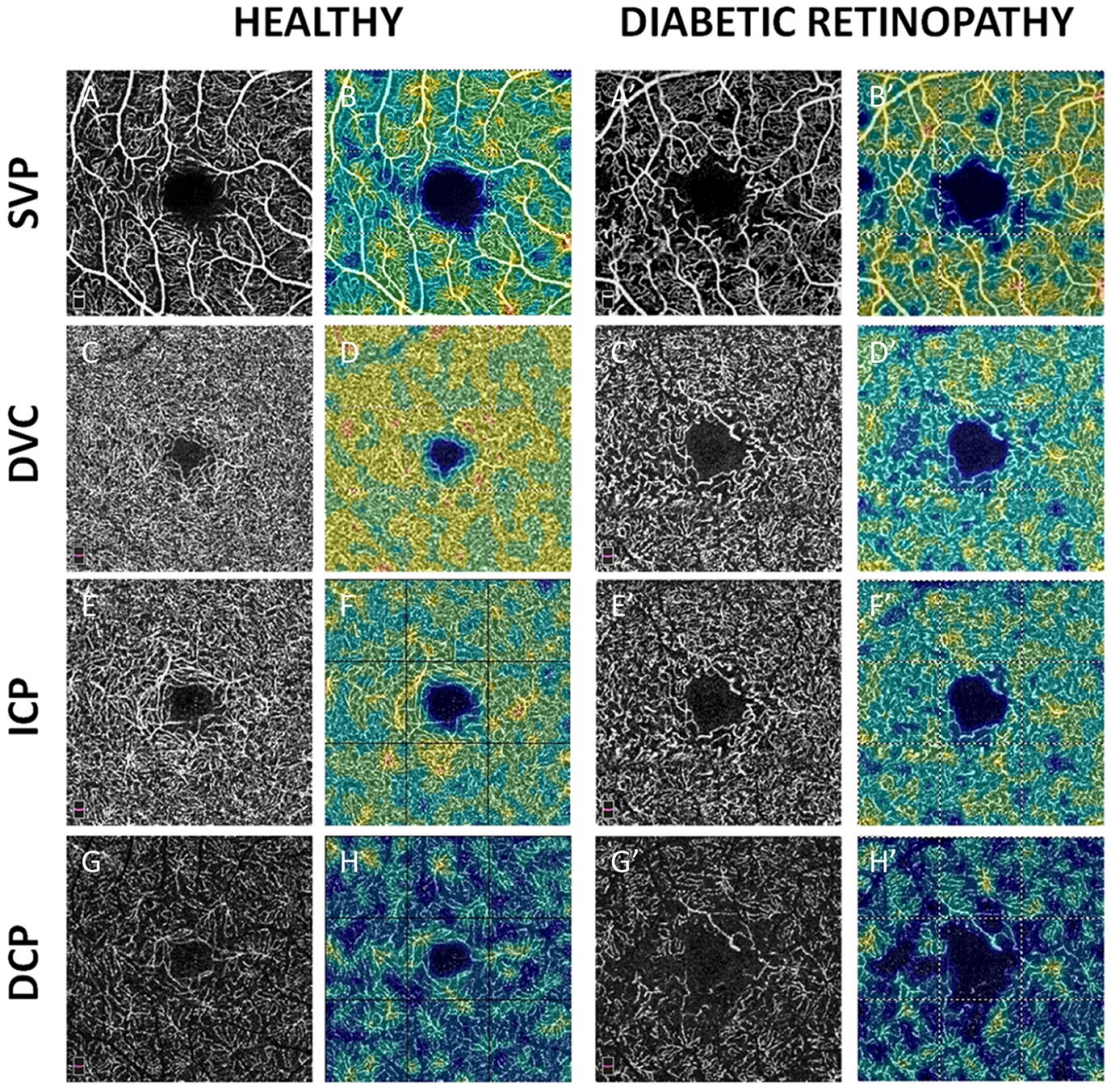
En-face OCTA angiograms of a healthy subject (left columns, A to H) and a T1D patient with severe NPDR (right columns, A’ to H’). Black and white scans (A, C, E, and G) represent OCTA angiograms. Color map scans (B, D, F, and H) represent color-coded VD in the corresponding OCTA angiograms. Warmer colors represent higher VD. A-A’ and B-B’: Superficial vascular plexus (SVP). C-C’ and D-D’: Deep vascular complex (DVC). E-E’ and F-F’: Intermediate capillary plexus (ICP). G-G’ and H-H’: deep capillary plexus (DCP). A rarefaction of the capillary meshwork on all OCTA angiograms was observed in the eye with severe NPDR compared to the healthy eye.

OCTA VD maps were analyzed in parallel with B-scans displaying the flow signal. The superficial vascular plexus (SVP), the intermediate capillary plexus (ICP) and the deep capillary plexus (DCP) were analyzed separately as well as the deep vascular complex (DVC), according to Campbell et al [14] (Fig 1). The ICP and DCP slabs were respectively the inner and outer parts of the DVC. Of note, there was no overlap between each vascular slab.

The predefined boundaries provided by Optovue software were used for the SVP and the DVC analysis, while the ICP and DCP were manually segmented by adjusting the segmentation boundaries as previously described [10]. The SVP was comprised between the inner limiting membrane (ILM) and 9 μm above the junction between the inner plexiform layer and the inner nuclear layer (IPL-INL), while the DVC was comprised between 9 μm above the IPL-INL junction and 9 μm below the outer plexiform layer and outer nuclear layer (OPL-ONL) junction. The ICP boundaries were set between 9 μm above the IPL-INL junction and 6 μm below the INL-OPL junction. The DCP boundaries were set between 6 μm below the INL-OPL junction and 9 μm below the OPL-ONL junction. The SVP slab therefore included the nerve fiber layer (NFL), the ganglion cell layer (GCL) and the major part of the IPL while the DVC slab included the outer part of the IPL, the INL, the OPL and the inner part of the ONL.

Automated VD was calculated using Phase 7 AngioAnalytic software in the SVP and the DVC, and VD of the customized ICP and DCP was analyzed using a research version of this software (Beta version 2016.200.0.37). VD and retinal thickness values were recorded for the parafoveal area (an annulus of 3.0-mm diameter around the central 1-mm diameter ring) and its sectors. The FAZ area and perimeter, the foveal acircularity index (AI), and the foveal VD 300 (FD-300) were also measured.

### Retinal layer thickness analysis on structural optical coherence tomography

The thickness of retinal layers was measured on the structural map corresponding to the 3x3-mm OCTA map acquired simultaneously with the VD map. The OCT software automatically segments the retinal layers and provides retinal thickness values within 10 predefined slabs, from the inner limiting membrane (ILM) to the Bruch’s membrane. In this study, three predefined slabs were evaluated: the ILM-RPE (total retinal thickness), the ILM-IPL (inner retinal thickness) and the INL-OPL (intermediate retinal thickness).

## Concordance between retinal vessel complexes and retinal layer thickness

Concordance between the retinal slab thickness and the VD in the corresponding retinal layers was evaluated as previously described [10]. In particular, concordances were tested between the ILM-IPL slab and the SVP and between the INL-OPL slab and the DVC. Capillary plexus projections are slightly offset compared to the corresponding retinal layers on structural OCT.

All structural B-scans, and B-scans with flow overlay were scrolled and controlled by two experts for the correctness of automated layer segmentation, the absence of intraretinal cysts, as well as for foveal avascular zone (FAZ) delineation. In case of segmentation errors, manual corrections were performed by the examiners and then evaluated by a third expert. The case was included in the analysis only if segmentation errors involved less than 5% of the total scan area (i.e., 15 B-scans). Eyes with B-scan tilting were excluded and particular attention was paid to the sided visibility of Henle’s fibers that prevented a correct delineation of the OPL and ONL [15].

From an initial cohort of 35 consecutive T1D patients diagnosed with severe NPDR or PDR without DME, 18 patients were excluded because they did not meet all inclusion criteria, with 10 patients excluded due to segmentation errors >5%, mainly localized at the INL-OPL interface. In total, 17 eyes of 17 patients were included in the analysis and compared to a cohort of 17 eyes of 17 age-matched healthy subjects.

### Statistical analysis

Continuous variables were checked to meet the normality conditions of the Shapiro-Wilk test. A parametric t-test or a non-parametric Mann-Whitney test was used when deemed necessary to compare the analysed variables between groups. Statistical analysis was performed using IBM SPSS Statistics V.24. Results are presented as the mean ± standard deviation (SD) or as the median with range for continuous variables, and as proportions (%) for categorical variables. A Pearson correlation coefficient was used to explore correlations between variables. P-values ≤0.05 were considered statistically significant.

## Results

### Patient characteristics

The mean age of the 17 T1D patients (10 men, 7 women) was 36.4 ± 6.8 years (range 23–46) and that of the 17 healthy subjects (10 men, 7 women) was 35.9 ± 7.2 years (range 25–51). The mean BCVA was 0.06 ± 0.09 logMAR (range 0–0.3) in T1D patients and 0.0 ±0.0 logMAR in healthy subjects.

In the T1D group, the mean disease duration was 21.4 ± 6.8 years (range 11–35), the mean HbA1c level was 8.5 ± 1.7% (range 6.5–12); 9 eyes had NPDR and 11 eyes previously underwent PRP.

### Quantitative analysis

OCTA SSI and QI were slightly lower in the T1D group (76.5 ± 5.4 and 8.2 ± 0.6, respectively) compared to healthy subjects (79.4 ± 4.3 and 8.5 ± 0.5, respectively), although the difference was not significant (p = 0.09 and 0.08, respectively).

**Fig 2:**
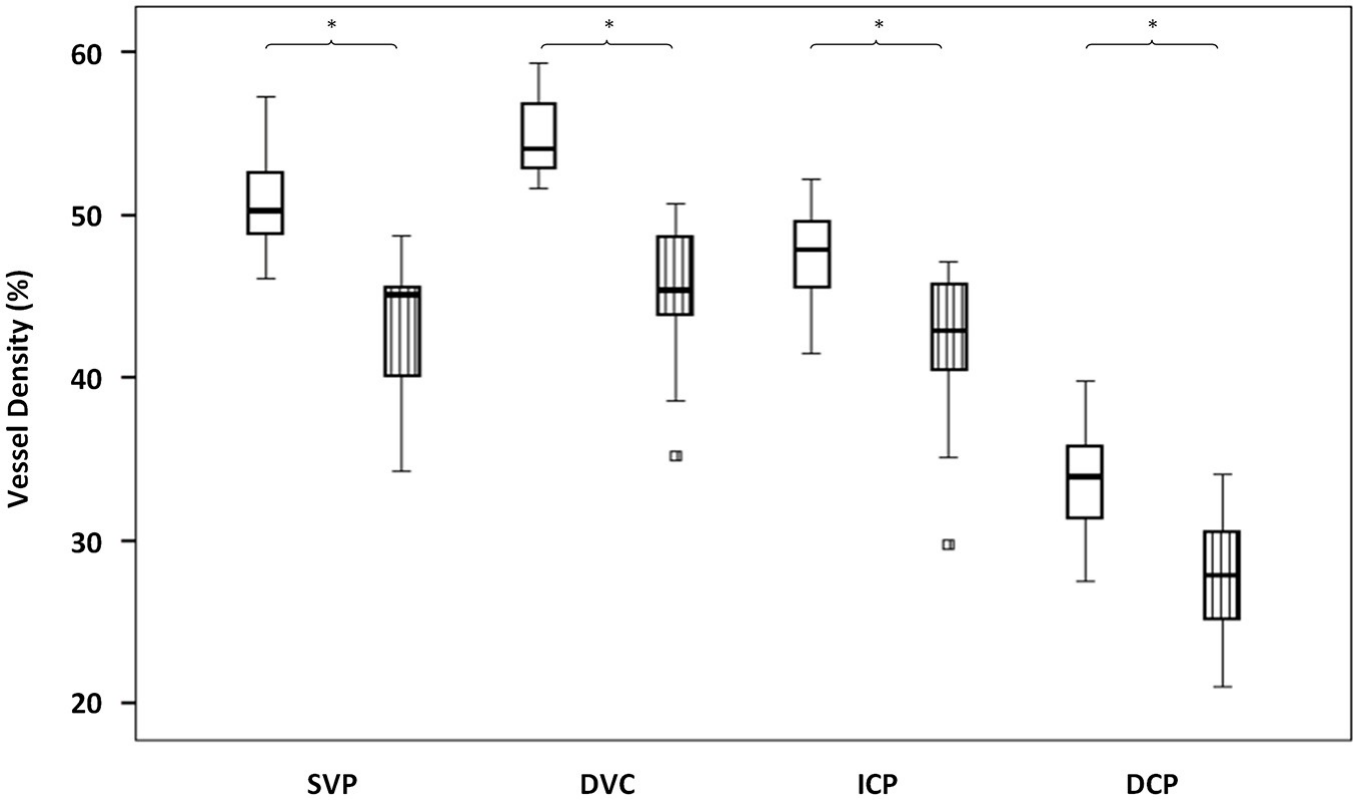
Parafoveal VD box-plots of the retinal vascular plexuses in the two groups. Healthy subjects: blank box-plots; diabetic retinopathy (DR) patients: box-plots with vertical stripes. VD (%) is represented on the y axis. Superficial vascular plexus (SVP), deep vascular complex (DVC), intermediate capillary plexus (ICP) and deep capillary plexus (DCP) are presented on the x axis. Asterisks over braces indicate a significant difference (p <0.05) between healthy subjects and DR patients in the corresponding plexus. Squares represent outside values. Upper and lower whiskers, respectively, represent the upper and lower adjacent values. Upper and lower box margins represent the 25th and 75th percentiles. The black horizontal line inside the box is the median value.

VD data are presented in Table 1 and Fig 2, retinal thickness data are presented in Table 1.

**Table 1:** Parafoveal vessel density (VD), foveal vascular zone (FAZ) and parafoveal retinal thickness (RT) mean values for the two groups. Values are expressed as mean ± standard deviation.

		Healthy eyes (n = 17)	DMT1 eyes (n = 17)	p-value
Parafoveal VD (%)	SVP	50.7 ± 2.7	42.8 ± 4.5	<0.001
	DVC	54.7 ± 2.4	45.4 ± 4.0	<0.001
	ICP	47.3 ± 3.0	42.1 ± 4.5	0.001
	DCP	33.8 ± 3.5	27.8 ± 3.7	<0.001
FAZ	Area (mm^2^)	0.23 ± 0.07	0.39 ± 0.17	0.003
	Perimeter (mm)	1.91 ± 0.29	2.74 ± 0.91	0.001
	AI	1.14 ± 0.05	1.24 ± 0.15	0.003
	FD-300 (%)	52.2 ± 2.5	46.4 ± 3.9	<0.001
Parafoveal RT (μm)	ILM-RPE	336.8 ± 11.1	328.4 ± 19.9	0.26
	ILM-IPL	118.4 ± 6.6	110.1 ± 9.9	0.04
	INL-OPL	70.2 ± 4.1	73.0 ± 8.3	0.39

**Fig 3:**
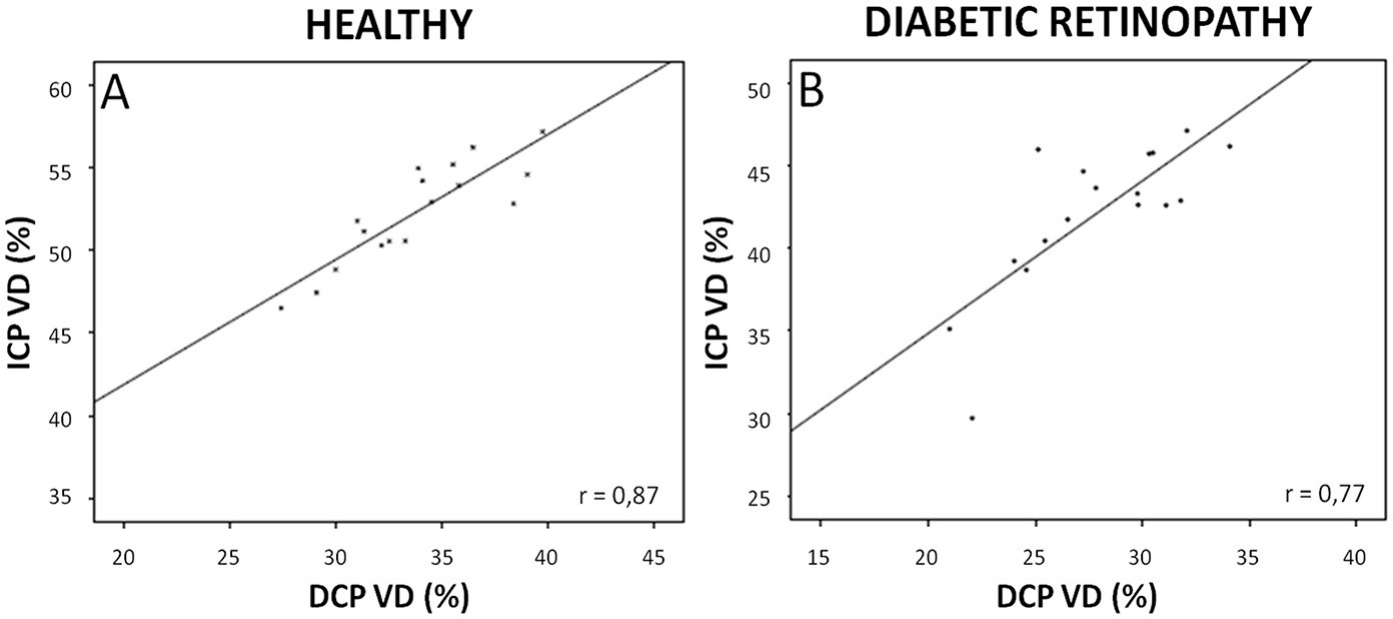
Correlations between vessel density in the intermediate and deep capillary plexuses. A and B: Scatter-plots with solid line showing the relationships between the parafoveal VD (%) in the intermediate capillary plexus (ICP, y axis) and in the deep capillary plexus (DCP, x axis) in healthy subjects (A) and diabetic retinopathy patients (B). Significant correlations (all p <0.01) were observed between the VD in the ICP and in the DCP in both healthy subjects and T1D patients. r = Pearson correlation coefficient.

In brief, compared to healthy subjects, the parafoveal VD was significantly reduced in T1D patients in the three retinal plexuses (all p <0.05). The decrease in VD was more pronounced but did not reach significance in the DCP (-17.6%) compared to the SVP (-15.5%), the ICP (-11.0%) and the DVC (-17.1%). The VD in the ICP positively and significantly correlated with the VD in the DCP in both groups (Fig 3; both p <0.001, r = 0.77 for T1D patients and 0.87 for healthy subjects).

**Fig 4:**
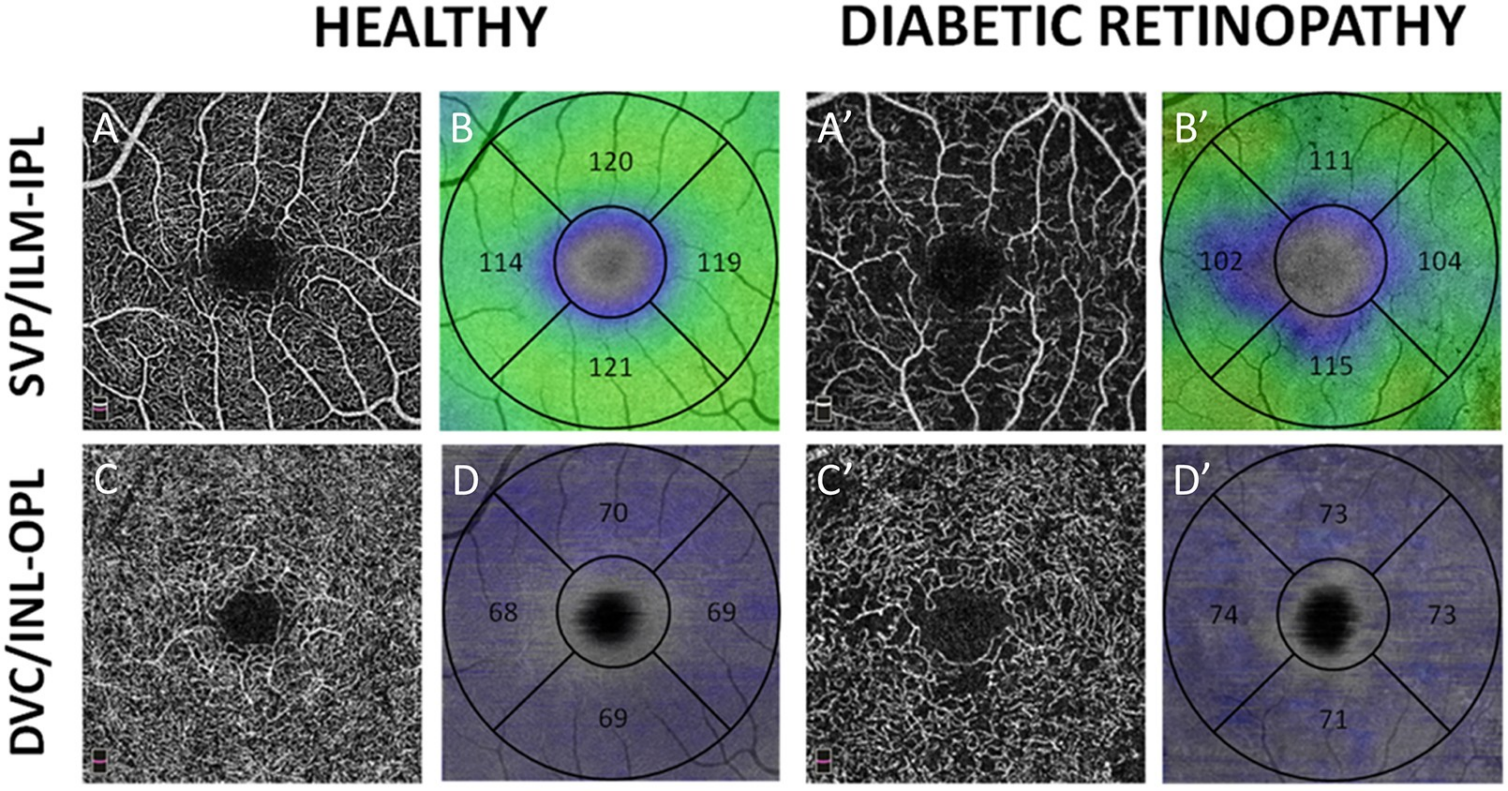
En-face OCTA angiograms (black and white) and retinal thickness maps (color-coded maps) of a healthy subject (left columns, A to D) and a T1D patient (right columns, A’ to D’). A-A’: OCTA angiograms of the superficial vascular plexus (SVP); B-B’: color-coded maps of the inner retinal thickness (from the inner limiting membrane, ILM, to the border between the inner plexiform layer, IPL, and the inner nuclear layer, INL). C-C’: OCTA angiograms of the deep vascular complex (DVC); D-D’: color-coded maps of the intermediate retinal thickness (including the INL and the outer plexiform layer). Thickness values in micron are reported from each quadrant of the parafoveal area within the color-coded maps.

The inner retinal slab (i.e. from the ILM to the IPL-INL border) in the T1D group was significantly thinner than in healthy eyes (p = 0.04). On the opposite, there was no significant difference in total (ILM-RPE) and intermediate (INL-OPL) RT between diabetic and healthy eyes (Fig 4).

Inner retinal thickness maps present areas of decreased thickness in DR patients compared to healthy subjects, in particular around the foveal avascular zone and correspond well to areas of capillary drop-out in the SVP. On the contrary, although the color pattern shows a higher irregularity in T1D patients, no obvious changes in intermediate retinal thickness are observed between DR patients and healthy subjects.

**Fig 5:**
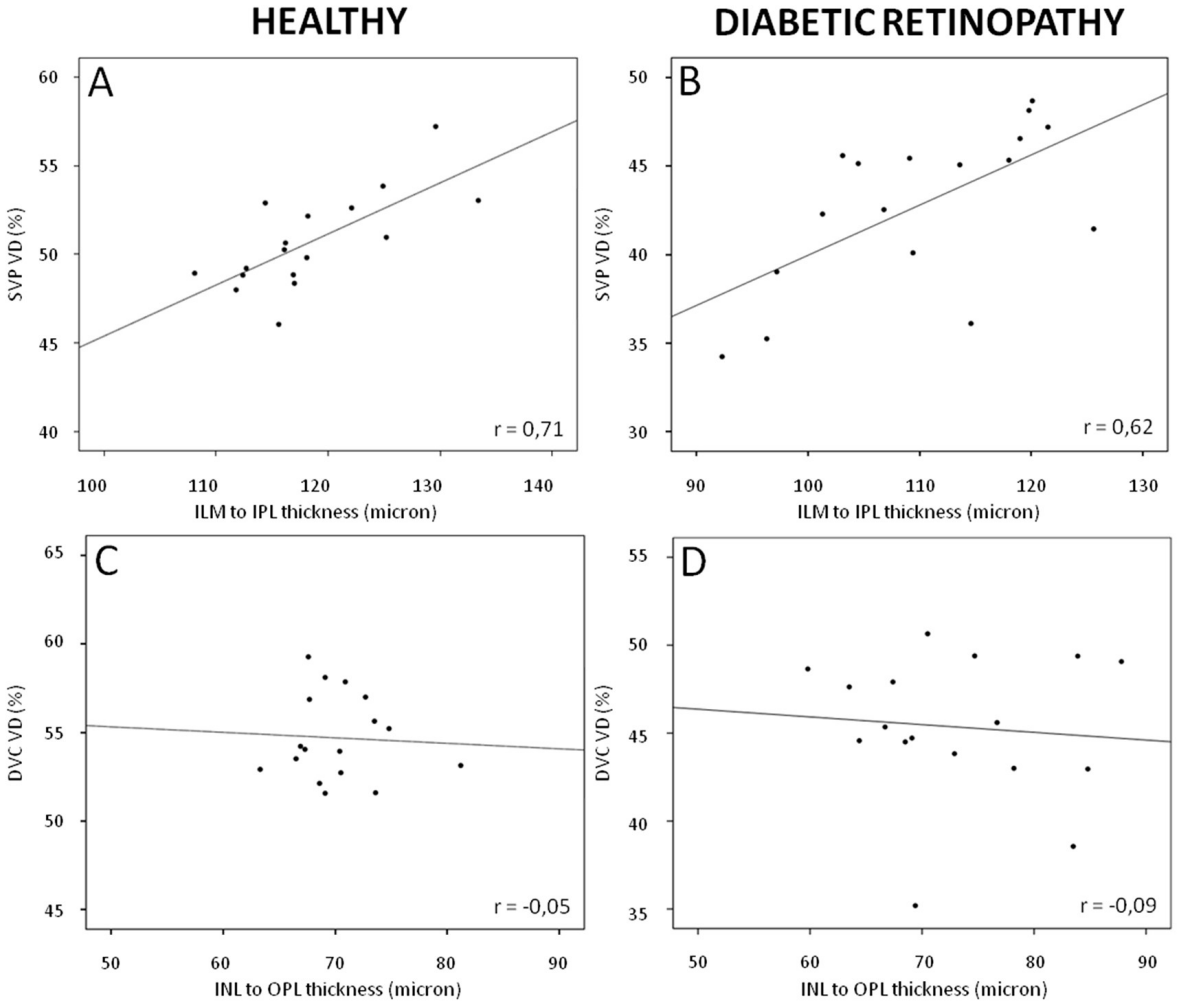
Correlations between vessel density (VD) and retinal thickness. Scatter-plots with solid line showing the relationships between the parafoveal vessel density (VD, %; y axis) and the retinal thickness (micron; x axis) in healthy subjects (A,C) and in diabetic retinopathy (DR) patients (B,D). A and B: significant correlations (all p <0.01) were observed between the VD in the superficial vascular plexus (SVP) and the inner retinal thickness (from the inner limiting membrane, ILM, to the border between the inner plexiform layer, IPL, and the inner nuclear layer, INL) in both healthy subjects (A) and DR (B) patients. C and D: no correlations were observed between the VD in the deep vascular complex (DVC) and the intermediate retinal thickness (including the INL and the outer plexiform layer) in both healthy subjects (C) and DR (D) patients. r = Pearson correlation coefficient.

There was a positive and significant correlation between the VD of the SVP and the thickness of the ILM-IPL slab in the eyes of healthy subjects (R = 0.71, p = 0.001). The VD of the SVP and the thickness of the ILM-IPL slab in T1D eyes were lower than in healthy eyes but remained significantly correlated (R = 0.62, p = 0.008). On the contrary, there was no significant correlation between the VD of the DVC and the thickness of the INL-OPL slab both in healthy eyes and in T1D eyes (R = -0.05, p = 0.84 and R = -0.09, p = 0.73, respectively). Correlation graphs are shown in Fig 5.

### Qualitative analysis

**Fig 6:**
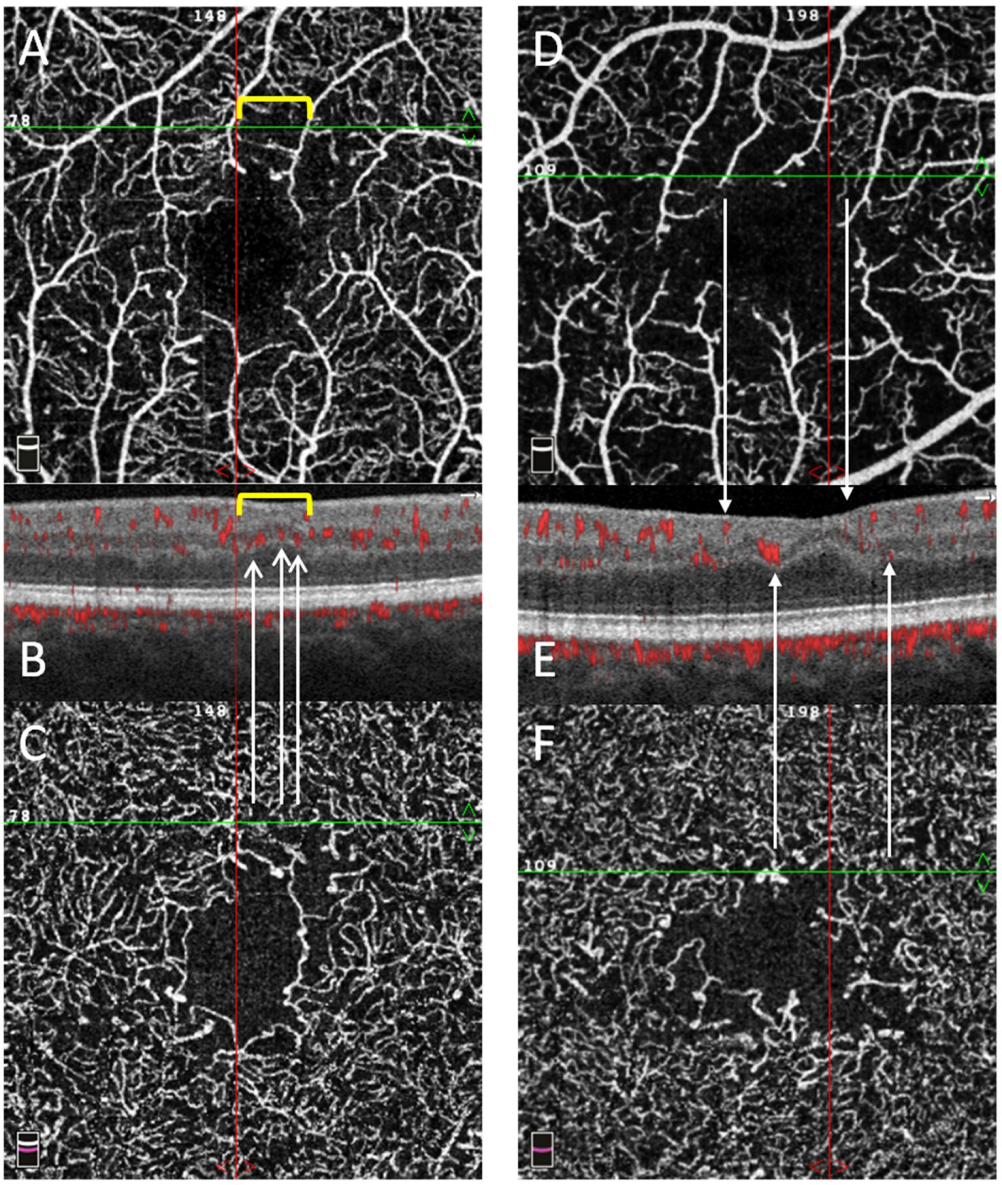
En-face OCTA angiograms and structural B-scans of two T1D patients. En-face OCTA angiograms of the superficial vascular plexus (SVP, A and D) and deep vascular complex (DVC, C and F) of two T1D patients with the corresponding structural B-scan with angio-overlay (B and E), both passing at the green line shown on the en-face OCTA angiograms. Different patterns of capillary drop-out are seen among the plexuses. In box A, an area of capillary drop-out in the SVP is delimited by the yellow bracket and corresponds to an area of inner retinal thinning and no flow signal in B. In the DVC, the flow signal is visible in the same area as in A, with visible capillaries in both boxes C and B (white arrows indicate red spots on the angio-overlay B-scan). In box D, two arrows from the SVP indicate two bigger vessels with no flow signal between them and a corresponding retinal thinning in E. In the DVC, the flow signal is visible in the same area as in D, with visible capillaries in both boxes F and E (white arrows indicate red spots on the angio-overlay B-scan). The inner nuclear layer and outer plexiform layer show an irregular wavy pattern on both boxes B and E.

In diabetic eyes, the pattern of capillary drop-out in the SVP differed from that observed in the DVC. In the SVP, well delineated polygonal areas of capillary drop-out were present between the distal arterioles and venules, especially around the fovea (Fig 6).

**Fig 7:**
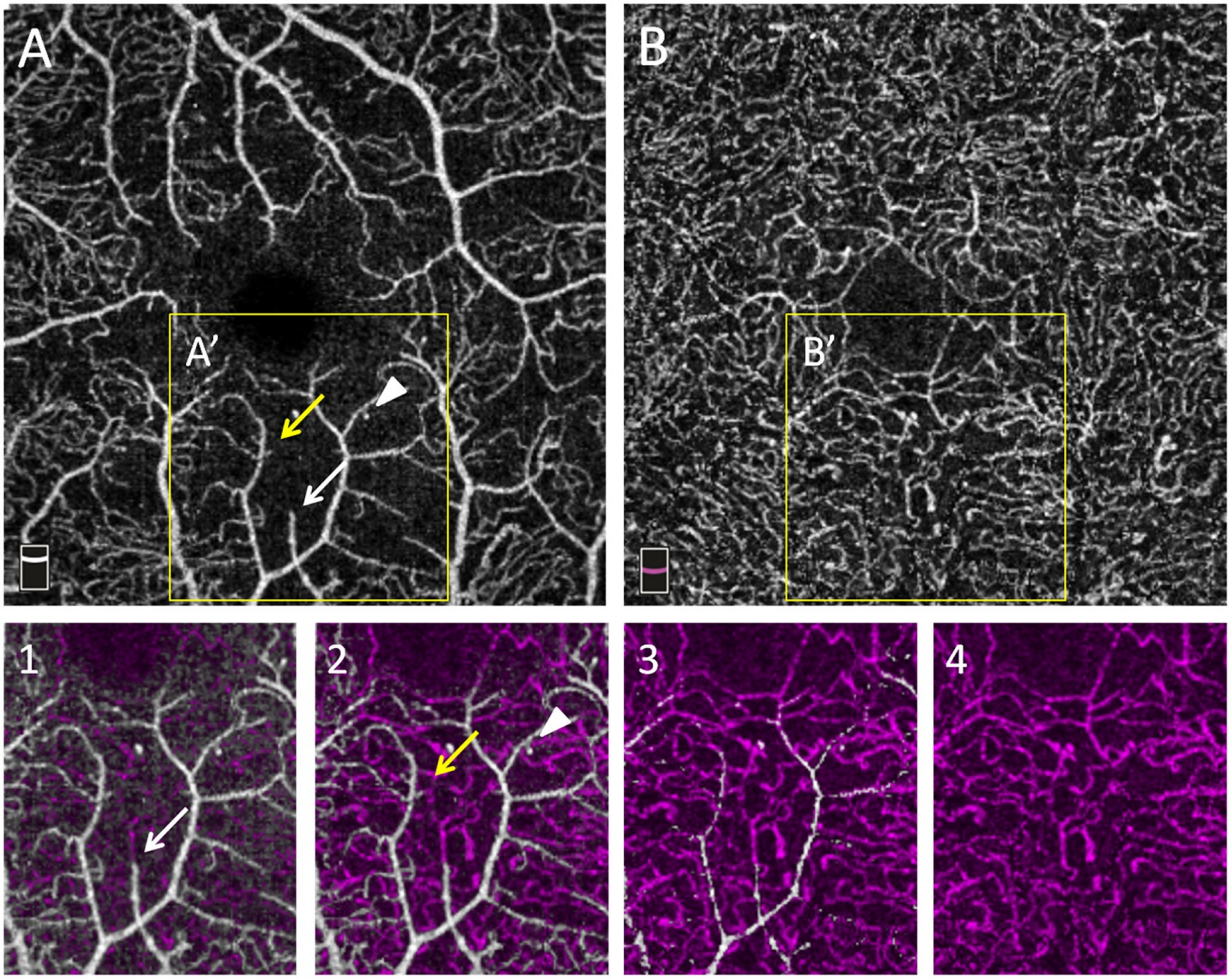
En-face OCTA angiograms of vascular plexuses in a T1D patient. En-face OCTA angiograms of the superficial vascular plexus (SVP, A) and deep vascular complex (DVC, B) in severe NPDR. A’ and B’ represent the same areas magnified in boxes 1,2,3,4. Boxes 1,2,3,4 represent consecutive color-coded OCTA slabs with the same thickness acquired at increasing depth from the SVP to the DVC. White vessels arise from the SVP while purple vessels arise from the DVC. Arrows (white and yellow) and the white arrow-head indicate residual capillaries in an area of capillary drop-out in the SVP (A) connecting with a denser capillary network in the DVC.

In the DVC, the reduction in capillary density was more diffuse: there were less polygonal areas of capillary drop-out, although globally, the reduction in VD in the DVC was similar to that found in the SVP. This is illustrated in several cases, in which there was some residual circulation in the DVC, below an area of capillary drop-out in the SVP (Fig 7).

On structural OCT, B-scans showed that, in the SVP areas of capillary drop-out, the inner retina was focally thinner in T1D eyes than in healthy eyes, while the intermediate retinal layers (i.e. INL—OPL) showed an irregular and wavy pattern but no significant thinning (Fig 6). These findings had their counterpart on thickness maps corresponding to the ILM-IPL and INL-OPL slabs, respectively (Fig 4).

## Discussion

In this series, we studied the correlation between the OCTA VD of macular capillary plexuses in DR eyes without DME and the thickness of the corresponding retinal slabs. We showed that in these cases of DR, the VD decreased in all plexuses compared to eyes of age- and gender-matched healthy subjects (Table 1, Fig 2). Areas of capillary drop-out in the SVP corresponded to a thinning of the NFL+GCL+IPL slab, while a decreased VD in the DVC did not correspond to a significant decrease in INL+OPL slab thickness (Fig 4).

The VD decrease in DR and its worsening with DR severity have been well documented with OCTA, although the results based on the analysis of the deeper plexuses are discordant [16–18].

The evaluation of the deeper capillary networks may be challenging due to segmentation errors in cases of ME or DRIL that do not allow the accurate delineation of the plexuses, projection artifacts from the SVP and a higher dependence on image quality [10,19,20]. To obtain more reliable results, we chose to analyze a cohort of T1D patients with severe DR but without DME or DRIL, OCTA images of good quality, and images of the DVC with projection artifact removal. The qualitative analysis of en-face OCTA images showed that there are different patterns of capillary dropout between the SVP and the DVC (composed by ICP-DCP).

We found that although VD was decreased in all plexuses, the pattern of capillary drop-out differed between the SVP and the DVC (Fig 6). In the SVP, some non-perfusion areas (NPA) appeared as polygons of various size between the terminal arterioles and venules, with loss of the capillaries bridging an arteriole to a venule. In the same area, the DVC remained perfused in all cases but one, although less dense than normal, and probably fed by some remaining vertical capillaries arising from the overlying arterioles (Fig 7). The widely anastomotic organization of the DVC could mitigate the effect of decreased perfusion from the SVP, avoiding well-delineated patches of NPA. However, in case of a large NPA in the SVP, no capillary perfusion persisted in the underlying DVC. These findings suggest that the evaluation of both the SVP and DVC for the assessment of capillary non-perfusion in DR using OCTA may be important for an accurate assessment of macular perfusion. Differences in terms of NPA patterns have been previously described by Dodo et al [21], who did not find any correlation between the transverse length of the superficial and deep NPAs and by a qualitative analysis by Couturier et al showing different patterns of microvasculature alterations between the two capillary plexuses (superficial and deep) [22]. Onishi et al have also observed a similar VD decrease in the ICP and in the DCP which worsened with DR severity, and the significant positive correlation we observed between the VD in the ICP and the VD in the DCP supports these findings [23]. Nesper et al have recently observed different responses of the macular vascular plexuses to light stimuli, showing that the SVP significantly differs from the ICP and the DCP, supporting distinct neurovascular control mechanisms potentially related to different neuromodulators acting on each capillary plexus [24].

Moreover we obtained simultaneous information on capillary blood flow and retinal structure in a single OCTA acquisition, so that it was possible to evaluate whether a reduction in VD in the capillary plexuses resulted in any change in thickness of the corresponding retinal layers, i.e., the ILM-IPL slab for the SVP and the INL-OPL slab for the DVC.

In this study of eyes with advanced DR but without DME or DRIL, we observed a significant decrease in NFL-GCL-IPL thickness up to 9.3% in the parafoveal area in T1D patients (p <0.05), as previously reported by others [25,26]. On the opposite, no significant difference was observed in INL-OPL thickness or in INL thickness alone compared to healthy eyes. The qualitative analysis of structural B-scans showed that while distinct areas of retinal thinning corresponded well to NPA in the SVP, no obvious changes in thickness were observed in the INL-OPL complex (Fig 4). Nevertheless, the aspect of the INL and OPL visibly differed from that of healthy subjects, appearing more irregular and wavy, with an increased visibility of Henle’s fibers within the ONL, the shape of which was irregularly dentate as previously described [27]. This wavy aspect was mainly due to the irregular thickness of the overlying GCL (Fig 6).

The decrease in VD in the SVP significantly correlated with the decrease in inner retinal thickness in DR, a well-known finding in mild DR and which can result in DRIL in case of more severe capillary drop-out [6]. On the contrary, no correlation was observed between the decrease in VD in the DVC and the INL/OPL thickness (Fig 5). The reason for this discrepancy, also found in healthy eyes, is unclear [10].

It is known from the histological study by Snodderly et al. on normal monkey eyes that there is a significant correlation between changes in volume of the “upper” (or “vitread”) capillary network (corresponding to the SVP) and the NFL+GCL thickness on different specimens. However, there was no correlation between the volume of the “lower” (or “scleral”) capillary network (corresponding to the DVC) and the INL thickness [28]. Chan et al. have also evaluated capillary networks in eyes of human donors and have suggested that capillary network morphometry is coupled with neuronal demands but observed a mismatch between the capillary density location and morphometry and the neuronal activity [29]. More recently, we have analyzed healthy eyes using OCTA and found significant correlations between the VD in the SVP and the ILM-IPL thickness while no correlation was found between the VD in the DVC and the INL-OPL thickness in healthy subjects [10]. Surprisingly, in our series of DR eyes, the decrease in VD in the DVC, although at least as severe as in the SVP, did not result in any significant INL thinning.

These findings should be related to the current knowledge that the tissue level of Pa02 is especially low in the INL and OPL, which receive their O2 supply from the ICP and the DCP [30]. The avascular INL, due to its partial dependence on anaerobic metabolism, would be less demanding in oxygen [28,31–33]. On the contrary, the presence of a dense SVP in the GCL indicates a high rate of oxygen exchange at this level [34]. It is likely that the decrease in capillary perfusion in the GCL results in some degree of cellular loss, while the INL might better adapt to hypoxia.

Another explanation could be that the absence of correlation between the decrease in VD in the DVC and the INL/OPL thickness could be due to the relatively homogeneous decrease in VD in the DVC. Indeed the intercapillary distance, necessary to guarantee an optimal oxygen diffusion, has been reported to be of about 60–75 microns in the parafoveal area [35]. With higher values, adequate oxygenation could be prevented, and retinal remodeling due to cellular damage or death could coexist. In the DVC, where large polygonal patches of NPA are less frequent than in the SVP, probably due to its widely anastomotic nature [36,37], oxygen delivery to the INL/OPL could be relatively more preserved, explaining the discrepancies in retinal thickness changes.

This study has several limitations, including its retrospective design and the small size of the cohort of DR patients. However, the small sample size is due to the use of strict selection criteria in order to exclude potential confounders such as media opacities, ME and DRIL, to facilitate reliable comparisons with healthy subjects. Another limitation is the use of customized segmentation boundaries for the delineation of capillary plexuses, but this segmentation was also applied to a large cohort of healthy subjects to obtain robust normative data. Furthermore, the segmentation used to delineate the capillary plexuses was not precisely the same as that used to delineate the cellular layers in which they are immersed. However, this difference is due to the basic principle of OCTA, and we did our best to ensure that the capillary layers corresponded to their cellular layer. Because these patients had no ME, they did not undergo fluorescein angiography. Therefore, we cannot completely exclude that some minor leakage from the DVC could mask a real INL thinning even in the absence of retinal cyst. Patient axial length, that could potentially influence VD values, was not available but all included patients presented a refractive error comprised between -3 and +2 diopters. Finally, measuring VD could not be the best method to investigate the capillary drop-out. Some authors have recently found that intercapillary areas could represent more robust indicators of capillary drop-out in the SVP, showing that an increased number of areas correlates with DR severity [38,39]. However, due to the overestimation of the capillary diameter with OCTA, the measurement of intercapillary areas may not be good and no data were provided in the DVC.

In summary, in a cohort of T1D patients with severe NPDR or PDR, we observed different retinal structural changes associated with reduced VD: patchy polygonal NPAs in the SVP corresponded well to areas of inner retinal thinning. The mean decrease in inner retinal thickness correlated with the decrease in VD in the SVP. However, the INL/OPL retinal thickness did not differ from that found in healthy subjects although the VD in the DVC was also reduced. The individual reaction of a retinal cellular layer to decrease VD remains to be elucidated and prospective longitudinal studies are warranted to better understand the relationships between structural changes and visual function in DR.

## Supporting information

S1 Table Database used in the study. (XLSX)
